# The Identification of Runs of Homozygosity Gives a Focus on the Genetic Diversity and Adaptation of the “Charolais de Cuba” Cattle

**DOI:** 10.3390/ani10122233

**Published:** 2020-11-27

**Authors:** Yoel Rodríguez-Valera, Dominique Rocha, Michel Naves, Gilles Renand, Eliecer Pérez-Pineda, Yuliaxis Ramayo-Caldas, Sebastian E. Ramos-Onsins

**Affiliations:** 1 Faculty of Agricultural Sciences, University of Granma, Bayamo 95100, Cuba; yrodriguezvalera@udg.co.cu (Y.R.-V.); eperezp@udg.co.cu (E.P.-P.); 2 GABI, INRAE, AgroParisTech, University Paris-Saclay, F-78350 Jouy-en-Josas, France; dominique.rocha@inrae.fr (D.R.); gilles.renand@inrae.fr (G.R.); 3 INRAE, URZ, 97170 Petit Bourg, Guadeloupe, France; michel.naves@inrae.fr; 4 Animal Breeding and Genetics Program, Institute for Research and Technology in Food and Agriculture (IRTA), Torre Marimon, 08140 Caldes de Montbui, Spain; 5 Plant and Animal Genomics, Centre of Research in Agricultural Genomics (CRAG) Consortium CSIC-IRTA-UAB-UB, Campus UAB, 08193 Bellaterra, Spain

**Keywords:** genomic inbreeding, effective population size, runs of homozygosity, candidate genes, tropical climate, adaptation

## Abstract

**Simple Summary:**

The Charolais de Cuba cattle is a tropical adapted breed founded in Cuba around 120 years ago from Charolais French specimens. Nowadays, it is still a closed breed and remains as a small population. In this work, we analyzed the inbreeding and diversity patterns, as well as the population size, of this recent adapted breed via a run of homozygosity (ROH) analysis. We found that the genomic inbreeding levels are higher in the Charolais de Cuba breed compared to French and British Charolais populations. Nevertheless, we detected that the effective population size experienced a very similar decline during the last century in the three Charolais populations studied. Finally, a number of regions with exceptional patterns of long homozygosity were identified in this breed, and these could be related to processes of adaptation to tropical conditions.

**Abstract:**

Inbreeding and effective population size (Ne) are fundamental indicators for the management and conservation of genetic diversity in populations. Genomic inbreeding gives accurate estimates of inbreeding, and the Ne determines the rate of the loss of genetic variation. The objective of this work was to study the distribution of runs of homozygosity (ROHs) in order to estimate genomic inbreeding (F_ROH_) and an effective population size using 38,789 Single Nucleotide Polymorphisms (SNPs) from the Illumina Bovine 50K BeadChip in 86 samples from populations of Charolais de Cuba (n = 40) cattle and to compare this information with French (n = 20) and British Charolais (n = 26) populations. In the Cuban, French, and British Charolais populations, the average estimated genomic inbreeding values using the F_ROH_ statistics were 5.7%, 3.4%, and 4%, respectively. The dispersion measured by variation coefficient was high at 43.9%, 37.0%, and 54.2%, respectively. The effective population size experienced a very similar decline during the last century in Charolais de Cuba (from 139 to 23 individuals), in French Charolais (from 142 to 12), and in British Charolais (from 145 to 14) for the ~20 last generations. However, the high variability found in the ROH indicators and F_ROH_ reveals an opportunity for maintaining the genetic diversity of this breed with an adequate mating strategy, which can be favored with the use of molecular markers. Moreover, the detected ROH were compared to previous results obtained on the detection of signatures of selection in the same breed. Some of the observed signatures were confirmed by the ROHs, emphasizing the process of adaptation to tropical climate experienced by the Charolais de Cuba population.

## 1. Introduction

As a result of importing superior breeds for meat production, which was the main objective before 1959 for the Cuban livestock industry, Charolais sires and dams were imported directly from France to Cuba from 1900 until 1923. These animals were the ancestors of the current Charolais de Cuba breed. The main destination of these imported animals was the east of the country, exactly in Jiguaní (today Granma province) on the Farm San José del Retiro with a current census of 700 animals. The Charolais de Cuba (CHCU) breed was then created and became adapted to tropical conditions. Other subsequent imports of Charolais animals were made in 1944 from Mexico, in 1957 from Texas, and finally in 1969 from Canada [1]. Since then, no new animals have been introduced to the CHCU population, which has evolved in a closed and isolated way, which implies the possibility that there has been a decrease in genetic diversity in this breed, as well as an increase in consanguinity.

Inbreeding is defined as the probability that two alleles at the same locus are identical by descent from a common ancestor [2]. The increase in inbreeding results in an increase in the frequency of homozygous genotypes, so this indicator can be used as an estimator of inbreeding. However, alleles that are identical by descent (IBD) and by state (IBS) cannot be differentiated, and both could be included in the same indicator. Runs of homozygosity (ROH), which are continuous and uninterrupted regions of DNA sequences without heterozygosity in the diploid state [3], have been proposed to solve this problem. ROHs allow for the distinguishing of these differences in many cases, and have been analyzed in several species [4,5,6].

In finite-size populations, inbreeding produces changes in genotypic frequencies, increasing homozygosity at expenses of heterozygosity, while allelic frequencies remain constant [7]. The presence of autozygosity is a consequence of individuals with a common ancestor that transmitted an identical segment of chromosome to their progeny, resulting in homozygous regions and an increase in the number of ROHs [8].

The concept of genomic inbreeding was recently introduced [9] in order to obtain a direct and accurate estimate of inbreeding. Genomic inbreeding is measured by F_ROH_, defined as the proportion of the (autosomal) genome in homozygous state while assuming that homozygous regions (ROHs) of the genome could only be the result of inbreeding, resulting in autozygosity [4]. The existence of medium to high correlation between F_ROH_ and other inbreeding indices suggests that the levels of autozygosity derived from ROHs can be used as estimators of individual inbreeding [10].

ROHs are more common in regions of high linkage disequilibrium (LD) and low percentage of recombination [3]. For example, in a study at the genomic level in individuals from human European populations [11], a non-uniform distribution of ROHs was detected, with almost identical patterns in all the studied populations. This structure could not only be explained by a reduced genetic diversity (genetic diversity explained only 7% of the ROH variation), a localized heterozygosity deficit, or a regional increase in LD. Historical and demographic events significantly modulated ROH patterns, detecting a geographic decline associated with population density and mobility in European populations.

In addition to inbreeding, one of the most widely used indicators to assess the genetic diversity of a population is its effective population size (Ne) [12,13,14,15,16], which is defined as the size of an ideal population that could have the same amount of genetic drift or the same inbreeding as the population under consideration [17]. It is an important parameter in quantitative and population genetics and in evolutionary and conservation biology since it determines the rate of loss of genetic variation [18]. Maintaining genetic diversity within a population is accomplished by maximizing effective size (Ne) or equivalently minimizing the increase in inbreeding across generations with appropriate mating schemes [10].

A review of the main methods used to estimate Ne [19] includes those that use LD. In 1971, a simple method based on the assumption of two loci of a finite population—monoecious and diploid—with random mating, the absence of selection, and mutation was proposed [20]. In 1981, a method to estimate Ne was implemented for a population with random crossover between neutral genes at various polymorphic loci [21]. The efficiency of the method was found to increase as the sample size and the number of genes with high LD values increased. A procedure based on the multilocus LD of a homozygous chromosome segment (CSH) [21] showed less variability in this measure than using LD, and it was found that CSH over long distances reflects better estimates of recent Ne under the assumption of constant linear growth and no mutation. However, in natural populations, the assumption of a linear change in Ne is usually unrealistic, as usually occurs in a much more complex way [15]. Nevertheless, the very versatile algorithm included in the software SNeP v1.0 [14] is based on different Ne dynamics of population size change across time [13] and, thus, allows for the calculation of the expected value of the linkage disequilibrium (E (r2)) under almost all population dynamics [15].

The objective of this work was to calculate ROH values to estimate the genomic inbreeding and effective size of the CHCU breed before comparing this information with the values observed in French (CHF) and British Charolais (CHUK) populations. In addition, this study explored the relationship of the ROHs observed with previous results obtained in the same breed with signatures of selection [22].

## 2. Materials and Methods

### 2.1. Data Used and Marker Quality Controls

The analysis was performed on populations of CHCU (n = 40), CHF (n = 20), and CHUK (n = 26). The used data were obtained from previous studies using the Illumina Bovine SNP50 genotyping chip on CHUK [23], CHF [24], and CHCU [22]. A single genotype file was made with the three populations using Plink 1.9 [25]. The markers were filtered with the following parameters: missing genotypes (geno > 0.1), minor allele frequency (MAF < 0.01), and Hardy–Weinberg equilibrium (*p* < 0.01). SNPs that did not meet these criteria were excluded. Therefore, we retained 38,789 SNPs for the analysis of Ne estimation. LD pruning was additionally applied to eliminate SNPs with high LD values that were not really IBD, as previously recommended [25], thus leaving a final number of 33,038 SNPs. After filtering, genomic inbreeding was estimated by a procedure recommended for evaluating genomic ROHs in small populations (small in relation to the perception of the risk that this population would become extinct [26]).

### 2.2. Detection of Runs of Homozygosity (ROH)

We used the same procedure implemented by the authors of [27] with the library DetectRuns [28] in R 3.4.2. Briefly, ROH detection was determined in sliding windows of 20 SNPs (--homozyg-window-snp 20), allowing for no more than one missing SNP (--homozyg-window-missing 1) and one heterozygous SNP per window (--homozyg-window-het 1). The minimum length of an ROH segment was 4 Mb (--homozyg-kb 4000), as recommended when using the Illumina Bovine 50K BeadChip [29]. The minimum SNP density was 1 SNP per 100 kb (--homozyg-density 1/100), and the maximum gap between two consecutive SNPs was 1000 kb (--homozyg-gap 10^6^ bp). Finally, the rate in which an SNP was included in the total of sliding windows was at least 0.05 (--homozyg-window-threshold 0.05). ROHs were classified into three classes according to their size: 4–8, 8–16, and >16 Mb, as previously reported [5,26,29,30].

### 2.3. Estimation of Genomic Inbreeding

The parameter used to calculate genomic inbreeding was the F_ROH_ through the formula F_ROH_ = ΣL_ROH_/L_AUTOSOME_ (proportion of the genome in ROHs over the total genome of 2,504,168,970 bp covered by SNPs) [4]. The estimation, summary, and graphs of F_ROH_ were made using the aforementioned DetectRuns R library. The average size of ROH (MN_ROH_), defined as the average of the size of each ROH segment (L_ROH_), and the sum of all ROH segments per animal (S_ROH_) were estimated. Furthermore, the F_ROH_ by chromosomes (BTA) (F_ROH_BTA_) was estimated similarly, using the formula:

F_ROH_BTA_ = ΣL_ROH_BTA_/L_BTA_ (1)

where ΣL_ROH_BTA_ is the sum of the size of each ROH segment per BTA and L_BTA_ is the size of each BTA covered by SNPs [6].

### 2.4. Estimation of Effective Population Size

The estimation of the recent demographic history of the three populations was calculated through the trend of the effective size of the population (Ne) using SneP v1.1 [14]. This software estimates the effective population size through the following equation [13]:

(2) $$N_{T\left(t\right)}={\left(4f\left(c_{t}\right)\right)}^{-1}\left(E{\left[r_{adj}^{2}|c_{t}\right]}^{-1}-\propto \right)$$

where *N_T_*_(*t*)_ is the effective size of the population in t past generations, equivalent to *t =* (2*ƒ*(*c_t_*))^−1^ [31], *ƒ*(*c_t_*) is a function related to the recombination rate (which is approximately equal to *c_t_* for small recombination values and a second order function for larger values), *c_t_* is the recombination rate defined by a specific physical distance between markers, *r*^2^*_adj_* is the adjusted LD value for the sample size (40 animals for CHCU), and α is a correction for the occurrence of mutations. The proposed α values were 1, 2, or 2.2 [20,32,33].

This procedure is based on the relationships between the variance of the LD at adjacent SNPs and the effective size of the population in the presence of the mutation, which allows one to estimate recent and old Ne [34]. Thus, we studied the trend of the effective population size of the three populations, firstly in ~20 generations, that corresponded to the approximate time since the import of the French Charolais in Cuba [1,35]; and secondly, estimating the population size until ~35 generations, which approximately corresponds to the creation of the French Charolais Herd Book in 1842 [36].

A minimum allele frequency (MAF) ≥ 0.05 was used to avoid low frequency errors and to improve the Ne estimate of [13,27]. Other included options were sample size (40, 20, and 26 for CHCU, CHF, and CHUK, respectively), -mindist (2,400,000 and 1,400,000 pb) and -maxdist (50,000,000 and 2,400,000 pb—20 and from 21 to 35 past generations, respectively), which are the minimum and maximum distances to be analyzed between SNP pairs in bp.

### 2.5. Candidate Regions Responsible of the CHCU Adaptation to Tropical Conditions

The ROHs reported in this study were compared with regions under selection previously reported in the same CHCU population [22]. In addition, genes within the ROH intervals were annotated using the Map Viewer of National Center for Biotechnology Information (NCBI) (https://www.ncbi.nlm.nih.gov/mapview/) of the bovine genome assembly release UMD3.1.1. Gene ontology and pathway analyses were carried out using the Panther version 13.1 software tool [37] (http://pantherdb.org).

## 3. Results

### 3.1. Estimation of Runs of Homozygosity (ROH) and Genomic Inbreeding

After merging and performing the quality control of the data, we finally retained 33,038 SNPs for data analyses. In concordance with [29], we considered the definition of Regions of Homozygosity (ROHs) as the segments of homozygous genotypes greater than 4 Mb in length, with a minimum number of 20 SNPs; we allowed for at least one heterozygous SNP to estimate genomic inbreeding [26] in order to avoid overestimating the number of ROHs [29]. Note that the utilization of genotype markers does not allow for the detection of all existent variants, so the number of ROHs was possibly overestimated. We assessed the impact of Single Nucleotide Polymorphism (SNP) density and genotyping errors using the Illumina Bovine 50 SNP genotyping chip on ROH identification, and we revealed an abundance of small segments (overestimated the numbers of segments 1–4 Mb long), suggesting that it was not sensitive enough for the precise determination of small segments. However, it proved suitable for detecting segments longer than 4 Mb. In the current study, the values of F_ROH_ > 4 Mb are shown in Table 1. These values were found to be higher on average in CHCU (0.057), intermediate in CHUK (0.04), and lower in CHF (0.034), and they had medium-high levels of dispersion, with coefficients of variation of 0.44, 0.54, and 0.37 for Cuban, French and British Charolais, respectively.

Table 1 shows a decrease in F_ROH_ in the three populations as the segment size increased. In all cases, estimates of recent inbreeding values (F_ROH_ at larger sizes) were lower compared to estimates of ancestral inbreeding (F_ROH_ at smaller sizes). The CHCU breed showed the highest F_ROH_ values for the 4–8, 8–16, and >16 Mb classes compared to CHF and CHUK. An inversely proportional relationship between the F_ROH_ values and the size of the segments of each size interval was also observed. The distributions of the number of ROHs according to the different categories are shown in Table 2.

**Figure 1:**
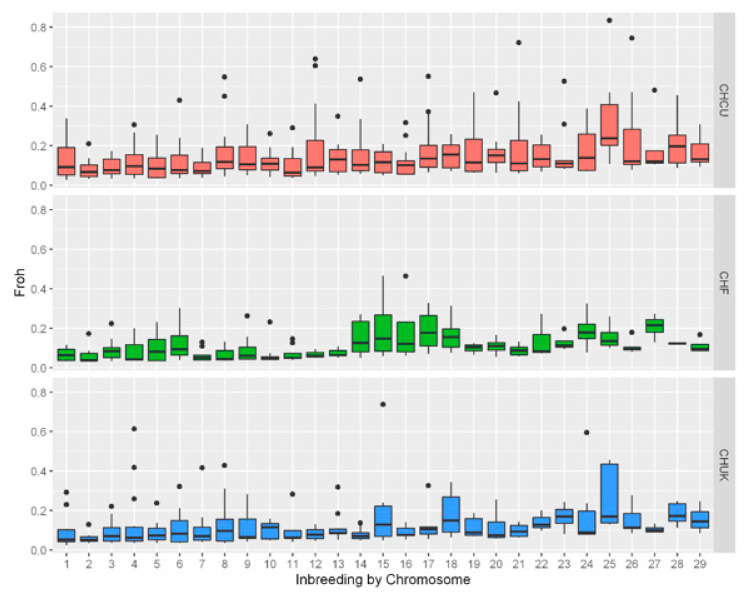
Distribution of genomic inbreeding estimates for each chromosome (F_ROH_BTA_), calculated as the proportion of BTA (F_ROH_ by chromosomes) in ROHs over the length of the BTA covered by the involved SNPs. The *x*-axis represents the chromosomes, and the *y*-axis represents F_ROH_ values in boxplot; black points are maximum values. ROH, run of homozygosity; CHF, French Charolais; CHCU, Charolais de Cuba; and CHUK, British Charolais.

For each chromosome, the F_ROH_BTA_ was determined as the proportion of BTA in ROHs over the length of the chromosome (BTA) covered by the studied SNPs (Figure 1 and Supplementary Materials Table S1). We observed a high variability within and between chromosomes (BTA), as well as high variability in the size and location of ROHs. Some animals showed high inbreeding values per chromosome; in CHF, F_ROH_BTA_ was >0.4 in BTA15 and 16; in CHCU, F_ROH_BTA_ exceeded 0.8 in BTA 25 and 0.5 in six other BTAs (8, 12, 14, 17, 21, and 26; see Figure 1); and in CHUK, three chromosomes (BTAs 4, 15, and 24) presented values > 0.5. In addition, there were overlaps between breeds with F_ROH_BTA_ values higher than 0.04: CHF and CHUK in BTA15 and CHCU and CHUK in BTA8 and BTA25. The distribution of the number, average size, and frequency of ROHs by chromosomes are shown in Table S2.

**Figure 2:**
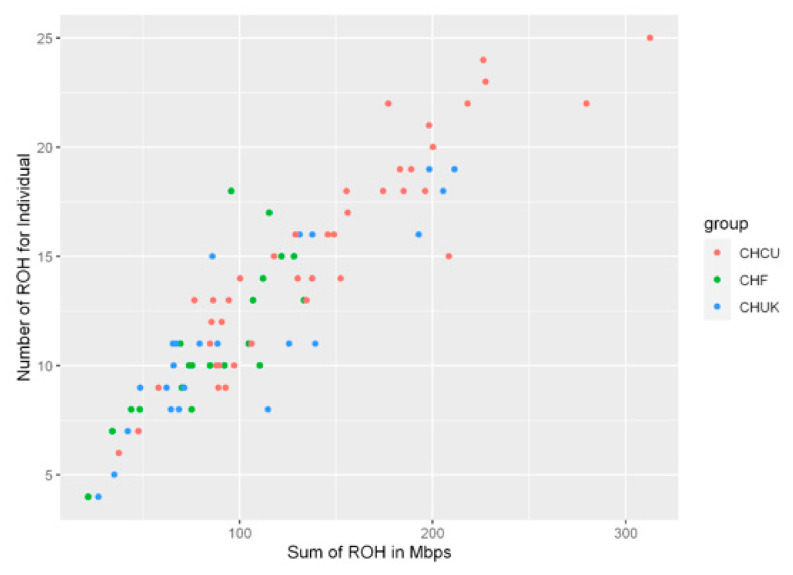
Relationship between the total number of runs of homozygosity (ROHs) >4 Mb and the total length (Mbps) of genome in such ROHs for individuals from the three analyzed Charolais populations. Each dot represents an individual.

The distribution of the number, average size, and frequency of ROHs per chromosome are shown in Table S2. We also estimated the average size of ROHs (Table 3) and the relationship between the total homozygous regions higher than 4 Mb with the size of each ROH in the three Charolais populations (Figure 2).

### 3.2. Estimation of Effective Populations Size

**Figure 3:**
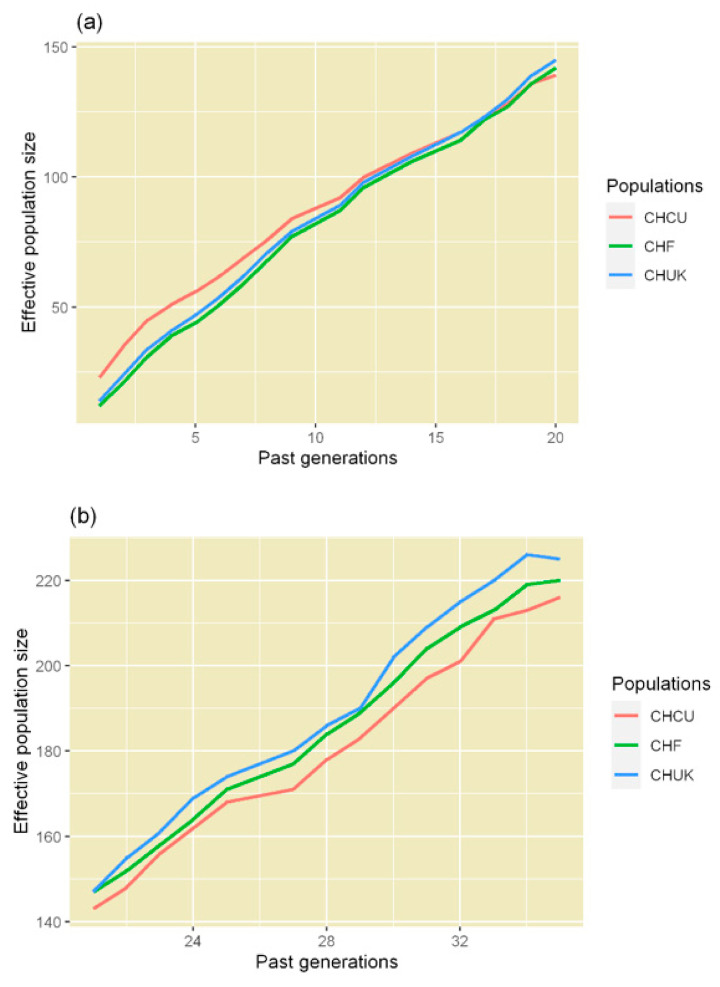
Effective size of three populations of the Charolais breed: (**a**) approximately 20 generations and (**b**) from 21 to 35 generations. Blue line, Charolais de Cuba; red line, French Charolais; and yellow line, British Charolais. *x*-axis: past generations; *y*-axis: effective population size.

During the recent period (Figure 3a), the observed trend showed a similar decrease in the three populations (CHCU: from 139 to 23; CHF: from 142 to 12; and CHUK: from 145 to 14). However, from generation 11 to the most recent generation, the CHCU population was found to have experienced a lower decrease than the others. Before this period (Figure 3b), the observed trend of the Ne was decreasing in a very similar way between the CHCU (from 216 to 143), CHF (from 220 to 146), and CHUK (from 225 to 147) as expected, because CHCU originated from ancestral individuals of these breeds.

### 3.3. Candidate Regions Responsible of the CHCU Adaptation to Tropical Conditions

We compared the patterns of homozygosity between CHCU and CHF with the Extended Haplotype Homozygosity (EHH) intervals reported in a previous study [22]. We observed overlaps in the regions of the genome that were previously classified as candidates for being affected by adaptation processes [22] by using the estimates of F_ROH_: 102 ROHs (12.30% of the total ROHs identified in the CHF and CHCU samples) distributed in the 29 BTAs that contained previously reported 50 regions (48.07% of the total). The F_ROH_ estimate fluctuated in these regions between a minimum of 0.002 and a maximum of 0.022.

## 4. Discussion

### 4.1. Limitations and Trade-Offs of the Analysis Based on Genotype Datasets and Small Sample Sizes

The use of genotypes obtained from genotyping SNP chips that are not specifically designed for the target population can bias the results because many exclusive variants would not be detected. This effect, named ascertainment bias, is a potential problem for the analysis of variability. Here, the effect of ascertainment bias (the SNP50 chip was designed mostly for the Holstein–Friesian breed) is apparently inexistent for the following reasons: (i) Charolais is a taurine breed that is closely related to the Holstein–Friesian breed in terms of the origin and evolution of the mutations [22]; (ii) the number of SNPs detected (38,789 before filtering for LD; see the Materials and Methods section) was on the order of found in other taurine populations, (e.g., Brown Swiss at 38,710, Pinzganer at 38,198, and Tyrol Grey at 42,997 for the same SNP chip [29]); (iii) here, small ROH regions that could be more affected by ascertainment bias (<4 Mb; see [29]) were excluded from the analysis; (iv) the comparative analysis of the distribution of F_ROH_ between populations was expected to be weakly affected by ascertainment bias because this effect would be very similar for all related Charolais populations. Nevertheless, the identified ROHs must be interpreted with caution, as they were only candidate regions for selection.

From a strict point of view, homozygosity is only accurately calculated with genome sequencing data, because it is the only way to unbiasedly estimate the levels of heterozygosity and, thus, homozygosity. The use of SNP chips, whatever considered density, eliminate private SNPs that only appear in one population. The use of medium- or high-density SNPs are approaches that detect a number of candidate regions effected by positive selection. Indeed, an optimal genotyping chip would satisfy the presence of separated SNPs with a good enough recombination rate to allow for separated segregation between them. This optimal separation is difficult to estimate and depends mainly on the population size, demography, and number of generations that mutations create. In the case of the Cuban Charolais population, which is a very recent population with small number of individuals, the chances for recombination are relatively low, suggesting that a medium SNP genotyping chip may be enough for such analyses. That is, in case of acting disparate evolutionary forces between sister populations (such as selective pressure), differences between populations would be observed because such forces would affect large genomic regions.

A large sample size would increase the number of detected variants but would also increase the effort required to search for samples in the field, as well as the genotyping cost. In this work, the optimal sample size necessary for such analysis was that which allowed for the detection of the effect of differential patterns in homozygosity. Here, we analyzed 40 samples of our target population (CHCU) plus 20 and 26 samples from the CHF and CHUK populations, respectively. Though this was a small sample size, the number of SNPs expected to be detected for a sample size of n = 700 diploid individuals (i.e., the entire CHCU population, which is around 200 times in relation to n = 40) is only 1.6 times larger than that expected to be detected using n = 40 (under standard assumptions [38]). In other words, although a small sample size was used and the results were limited, the study of a large sample in homozygosity analysis would not greatly increase the number of detected SNPs. Note that an analysis of homozygosity needs a very different experimental design from that in Genome Wide Association Studies (GWAS) studies, where a large number of samples is needed to achieve meaningful association results of the genome variants.

### 4.2. Estimation of Runs of Homozygosity (ROHs) and Genomic Inbreeding

Inbreeding is a highly variable measure between different cattle populations that largely depends on the isolation of the population, size, and genetic improvement programs used, among other factors [39]. Additionally, the number of SNPs, the density of the SNP chip, and the selection criteria for the used SNPs to determine the ROHs can have a huge effect on F_ROH_ values [40]. It was expected that CHCU would have higher LD as a result of their small size and isolation. This was observed (Table 1) using the global statistics of F_ROH_ and MN_ROH_, although we also observed that the LD determined by MN_ROH_ at long fragments (Table 2, MN_ROH_ > 16 Kb, affected by recent events) was higher in CHCU in relation to CHF but surprisingly lower compared to CHUK. This surprising result may be explained by an event of recent strong selection pressure in CHUK or by the contribution (although limited) of few animals from other breeds to CHCU [41].

A comparison of our results to the bibliography is presented in Table S3. Similar results in F_ROH_ values have been found in local cattle breeds in Italy [26,30], Spain [10], dual-purpose cattle in Austria [5], and US Holsteins [40]. These authors also pointed out the importance of mating strategies in maintaining genetic diversity in local breeds. Lower values have been found in local breeds in Africa [42], while higher values have been observed in Blanc Bleu Belge [43], Spanish Lidia [44], and the Gyr breed in Brazil, which was explained by a small Ne and a high LD value [45,46].

The estimates of F_ROH_ not only allowed for the measurement of individual autozygosity but also the distinguishing between ancient and recent inbreeding values. This explains why recombination events interrupt large chromosome segments. ROHs (~10 Mb) appear as a result of recent inbreeding (up to five past generations), while small ROHs (~1 Mb) may indicate a greater ancestral distance (up to 50 generations past) [4]. For example, an inversely proportional relationship of F_ROH_ and class segments was found when estimating F_ROH_ in Fleckvieh cattle from Austria [5]. In addition, similar results were observed when evaluating the autozygosity, effects of genotyping errors, and density of SNPs using high and medium density panels in the three following bovine breeds: Brown Swiss, Pinzgauer, and Tyrol Grey [29]. Other authors have also observed the same patterns for different populations [47,48].

We observed a high variability within and between chromosomes (BTA) in the size and location of ROHs, which was in agreement with previous studies performed in bovines [10,29,49].

### 4.3. Estimation of Effective Populations Size

The method used to estimate Ne uses the LD for this purpose. Several authors have found an inverse relationship between these two indicators when comparing them between various breeds of cattle [16,50,51]. In this same way, it has been reported [22] that the LD model between CHF and CHCU is lower in CHCU compared to CHF, with this decrease in LD observed between both breeds seen as a result of strong selection pressure in CHF (a consequence of the improvement programs of this breed in France) compared to the CHCU.

Some limitations in the estimation of Ne have been attributed to the used methodology [13]. Estimating the Ne variable at different times found results that caused certain doubts, especially for recent generations (T < 10) even for a corrected sample size. However, they indicated that methodology is highly sensitive to a value of α (correction for the occurrence of mutations). Finally, these authors advised being extremely careful, especially in terms of conservation, with decisions made based on estimates of Ne when it is assumed to vary. However, these limitations have been addressed in SneP according to its developers [14], although they clarified that the quantitative estimates are still highly dependent on the sample size, the type of estimation of the LD, and discrete interval processes.

The severe reduction of the estimated population size of CHCU can be explained by the founder effect related to the reduced number of sires that were exported to Cuba, as well as to the inbreeding created by the repeated use of these sires, which has been even more dramatic due to the selection scheme and artificial insemination that was massively introduced in Cuba in the 1960s [52]. The occurrence of a bottleneck in French Charolais cattle was previously detected by pedigree analysis by the authors of [53], who argued that it occurred due to the increase in the use of artificial insemination (AI) in the 1990s and the intensity of selection.

Estimates of an effective size smaller or similar to those found in CHCU were obtained in Chinese cattle breeds [16] in the north (Ne = 34), south (Ne = 27), southwest (Ne = 31), and the Wagyu breeds (Ne = 14), with the difference being that in those studies, the Ne values were determined for 1000 generations up to the most recent generation. These authors suggested that it was possible to use genomic selection programs adapted to breeds with a small effective size in these breeds. There is no evidence that new Charolais animals have been imported since 1969 to Cuba [1], so CHCU has a small degree of differentiation with its ancestor CHF (Fst = 0.0497) [22]. In addition, CHCU is a breed adapted to tropical conditions, so CHCU could generate an economic benefit. This represents a real possibility of extending its use through the flow of CHCU alleles to other latitudes, especially in the current context of global warming.

The existence of a high variance in the F_ROH_ indicators determined in CHCU (Figure 2) could also have been the result of possible crossbreeding events with animals of other breeds of Charolais [41], as explained by imports made from other countries [1], or the possible introgression of alleles of zebu origin [54]. To avoid the accumulation of deleterious variants that would seriously affect the fitness of a breed, it is recommended to have at least Ne = 50 sires per generation [55], which corresponds to a consanguinity rate of 1%. According to this, all populations in conservation plans should achieve this minimum as a first objective. In each conservation program, despite the existence of a bottleneck, as manifested in CHCU, attempts should be made to conserve genetic diversity. If the population is very small, as in the investigated case, each animal is extremely important because its loss has a great impact on the total size of the population [55].

### 4.4. Candidate Regions Responsible of the CHCU Adaptation to Tropical Conditions

CHCU animals have adapted well to the tropical conditions and have developed a tolerance to high temperatures [22]. The analysis of ROHs allowed us to detect large regions that may be affected by positive selection. These regions should be considered with caution, because there are many reasons to find autozygosity (e.g., a low density of the SNP genotyping chip, genetic drift caused by population reduction or other demographic causes, and regions containing low recombination or low mutation rates, in addition to selective sweeps). Furthermore, the detected candidate regions contain a large number of genes, so it was mostly expected to find genes with functions related to the causal process of interest for the researcher, although they are not involved in such a process [56].

In this work, we found several ROHs overlapping with the regions under selection, in concordance with [22]. A total of 1195 genes in CHF and 4086 genes in CHCU were in the ROHs. BTA5:17.363:33.013, BTA5:6.285:36.84, and BTA5:21.708:33.687 were found to overlap with BTA5:28.660:30.114 [22], and they were considered together as a unique selection signal. Many genes located within the selected regions in CHCU have already been linked to heat stress. For instance, *AQP5* is located in three ROHs, as also reported as putative selective sweeps by the authors of [22]. *AQP5* encodes a channel protein that selectively transports water through the plasma membrane of secretory and absorptive cells found, for example, in salivary or sweating glands [57].

Other genes, such as heat shock proteins (HSPs), play central role sin cytoprotection during heat stress by protecting the animal against hyperthermia, circulatory shock, and cerebral ischemia via the overexpression of HSPs [58]. The activation of these systems appears to be initiated at skin surface temperatures exceeding 35 °C as animals begin to store heat and rapidly increase evaporative heat loss mechanisms [59]. One potential cytoprotection system that can be present in CHCU is the activation of heat shock transcription factor 1 (*HSF1*). This gene coordinates thermal tolerance, and it was found in CHCU ROHs BTA14:1.464–14.362. In addition, we found that the heat shock protein-related gene *HSPB1* is located in ROH BTA25:34.446:42.851 in CHCU, and it encodes a small heat shock protein that functions as a molecular chaperone [60]. This ROH overlaps with another interesting region reported as a selective sweep by the authors of [22].

The importance of the *SLICK* locus in tropical adaptation has been previously shown [61]. A study (GWAS) of the Limonero animals using Illumina BovineHD genotypes from 20 *SLICK* and 53 non-*SLICK* individuals was made by the authors of [62]; they identified significant associations to markers in and around *PRLR* (prolactin receptor). Two SNP markers intragenic to *PRLR* presented genome-wide significance, namely rs42551770 (20:39104658, *p* = 2.51 × 10^−8^ bp) and rs137009256 (BTA 20:39110968, *p* = 1.67 × 10^−8^ bp). Later, the authors of [63] found PRLR genetic variants associated with both the undercoat and the topcoat that have the potential to be used for selection in order to breed for animals with shorter hair, thus leading to greater thermotolerance and potentially increasing production in hot and humid climates. Interestingly, we found *PRLR* annotated in ROHs BTA20:38.341–71.794 in CHCU.

*ACHE* is another gene potentially involved in thermotolerance because it has been found to be associated, for instance, with an acute heat stress response in chickens [64]. Interestingly, we also found *ITGA9*, which was found to be associated with heat tolerance in summer in a genome-wide association study for body temperature performed in US beef cattle [65]. This gene is located within ROH BTA22:4.113:12.47 and was also identified as a selective sweep by the authors of [22] on BTA22:10.477:11.159.

One study on the genetic diversity of 21 autochthonous cattle breeds from the whole Mediterranean basin performed genome-wide association analyses with covariables discriminating the different Mediterranean climate subtypes [66]; the authors of this work found various genes associated with climatic covariables that overlapped within the ROHs found in the current study: *PTPRF* (protein tyrosine phosphatase receptor Type F) on BTA3:90.368–117.379 in CHF; *NRG1* (neuregulin 1) on BTA27:15.885–28.268 in CHF, *OTUD7A* (OTU deubiquitinase 7A) on BTA21:2.75–53,659 in CHCU, *C5H12orf54* and *RACGAP1* (Rac GTPase activating protein 1) on BTA5:17.363–33.013, BTA5:6.285–36.84, and BTA5:21.708–33.687 in CHCU are associated with climatic covariables—particularly *ANTXR2* (anthrax toxin receptor 2) on BTA6:90,666–119,217 in CHCU, which is associated with both the annual mean temperature and THI (temperature humidity index), and *TRAM2* (translocation associated membrane protein 2) on BTA23:2.651–25.55, BTA23:19.069–25.553, and BTA23:20.348–25.553 in CHCU, which is associated with annual mean radiation and annual precipitation [66].

## 5. Conclusions

In this work, we estimated, for the first time, the genomic inbreeding value and effective size of the CHCU breed using a medium-sized SNP panel, and then we compared this information to that of two closely related European Charolais populations.

Genomic inbreeding levels were found to be higher in the CHCU breed compared to the CHF and CHUK populations. The genomic inbreeding patterns were in agreement with the results obtained with the estimates of the effective population size, with low estimated Ne values and a tendency to decrease along generations. However, the high variance found in the indicators of ROHs and F_ROH_ suggest an opportunity to maintain the genetic diversity of this population with an adequate mating strategy that, accompanied with the benefits of molecular tools, could guarantee a better management of this important genetic resource.

Finally, we found several ROHs in CHCU on BTA2, 5, 14, 25, 20, 22, 21, and 23 that might be associated with environmental adaptation traits.

## Figures and Tables

**Table 1:** Genomic inbreeding (F_ROH_) according to three intervals (Mb) in three Charolais populations. CHCU: Charolais de Cuba; CHF: French Charolais; CHUK: British Charolais.

Breeds	4–8 Mb F_ROH_ (±SD)	8–16 Mb F_ROH_ (±SD)	>16 Mb F_ROH_ (±SD)
**CHF**	0.034 (0.012)	0.017 (0.011)	0.007 (0.007)
**CHCU**	0.057 (0.025)	0.037 (0.022)	0.016 (0.016)
**CHUK**	0.040 (0.022)	0.023 (0.019)	0.011 (0.014)

F_ROH_: Genomic inbreeding obtained from runs of homozygosity (ROHs) of 4–8, 8–16, and >16 Mb. ±SD: standard deviation.

**Table 2:** Number of runs of homozygosity (ROHs) by breed and interval in Mb.

Interval (Mb)	CHF			CHCU			CHUK		
	Number	Freq (%)	MN_ROH_ (Mb)	Number	Freq (%)	MN_ROH_ (Mb)	Number	Freq (%)	MN_ROH_ (Mb)
4–8	158	71.5	5.35	354	58.3	5.549	193	65.6	5.598
8–16	47	21.3	11.072	182	29.9	11.24	73	24.9	10.633
>16	16	7.2	22.195	72	11.8	23.634	28	9.5	27.377

Number: ROH number found in each class. Freq: percentage of ROHs in each class. MN_ROH_: average ROH size in Mb in each class.

**Table 3:** Average size of ROH (MN_ROH_) in three Charolais populations.

Populations	Class Mb	N_ROH_	MN_ROH_ (Mb)	±SD	CV %	IC −95%	IC +95%
**CHF**	4–8	158	5.34	1.08	20.28	5.18	5.52
	8–16	47	11.07	2.27	20.49	10.40	11.73
	>16	16	22.19	5.65	25.49	19.18	25.21
**CHCU**	4–8	354	5.54	1.14	20.69	5.42	5.67
	8–16	182	11.24	2.19	19.51	10.91	11.56
	>16	72	23.63	9.35	39.58	21.43	25.83
**CHUK**	4–8	193	5.60	1.14	20.37	5.44	5.76
	8–16	73	10.63	2.07	19.47	10.15	11.11
	>16	28	27.37	13.22	48.29	22.25	32.50

N_ROH_: Numbers of ROH. MN_ROH_ (Mb): average ROH size in Mb. ±SD: Standard deviation. CV (%): Coefficient of variation. IC −95%: Confidence interval; IC +95%: Confidence interval.

## Supplementary Materials

The following are available online at https://www.mdpi.com/2076-2615/10/12/2233/s1, Table S1: Statistic summary the F_ROH_BTA_ per chromosome in three Charolais cattle populations. Table S2: Distribution of the number and size of runs of homozygosity (ROH) by populations and chromosomes (BTA). Table S3: Value estimated of F_ROH_ in other cattle breeds.
