# Supplementary material for: The Identification of Runs of Homozygosity Gives a Focus on the Genetic Diversity and Adaptation of the “Charolais de Cuba” Cattle
Source: Animals (Basel). 2020 Nov 27;10(12):2233. doi: 10.3390/ani10122233 (PMC7760288; doi:10.3390/ani10122233)
Supplement: Supplementary file 1 [file animals-10-02233-s001.pdf]

Table S1: Statistic summary the F_{ROH_BTA} per chromosome in three Charolais cattle populations.

CHA						
BTA	F_{ROH_BTA}	±SD	Min	Max	Median	CV(%)
1	0.014	0.032	0.000	0.114	0.000	235.110
2	0.020	0.042	0.000	0.171	0.000	212.080
3	0.062	0.060	0.000	0.223	0.053	96.870
4	0.029	0.054	0.000	0.200	0.000	185.620
5	0.035	0.065	0.000	0.230	0.000	184.800
6	0.060	0.084	0.000	0.302	0.019	139.520
7	0.031	0.038	0.000	0.129	0.018	125.150
8	0.023	0.038	0.000	0.132	0.000	166.330
9	0.045	0.067	0.000	0.261	0.020	150.240
10	0.033	0.053	0.000	0.230	0.020	158.610
11	0.031	0.043	0.000	0.145	0.000	141.140
12	0.010	0.026	0.000	0.093	0.000	255.600
13	0.018	0.034	0.000	0.108	0.000	186.690
14	0.075	0.097	0.000	0.270	0.024	129.520
15	0.041	0.111	0.000	0.465	0.000	273.150
16	0.038	0.108	0.000	0.462	0.000	281.640
17	0.047	0.098	0.000	0.329	0.000	205.820
18	0.042	0.086	0.000	0.312	0.000	204.570
19	0.015	0.037	0.000	0.125	0.000	253.370
20	0.044	0.060	0.000	0.165	0.000	135.380
21	0.035	0.047	0.000	0.131	0.000	134.600
22	0.039	0.075	0.000	0.272	0.000	192.240
23	0.026	0.056	0.000	0.196	0.000	217.650
24	0.038	0.088	0.000	0.325	0.000	231.760
25	0.046	0.079	0.000	0.259	0.000	170.550
26	0.027	0.052	0.000	0.178	0.000	189.880
27	0.042	0.089	0.000	0.273	0.000	213.570
28	0.012	0.037	0.000	0.127	0.000	308.210
29	0.022	0.048	0.000	0.167	0.000	216.770
CHCU						
1	0.085	0.095	0.000	0.338	0.056	112.290
2	0.038	0.051	0.000	0.210	0.000	134.280
3	0.036	0.053	0.000	0.173	0.000	148.740
4	0.059	0.082	0.000	0.305	0.017	138.050
5	0.037	0.068	0.000	0.255	0.000	184.150
6	0.038	0.086	0.000	0.430	0.000	223.820
7	0.039	0.054	0.000	0.189	0.000	139.780
8	0.064	0.121	0.000	0.548	0.000	188.150
9	0.062	0.081	0.000	0.309	0.000	132.180
10	0.072	0.067	0.000	0.261	0.075	93.520
11	0.052	0.069	0.000	0.289	0.038	132.320
12	0.092	0.155	0.000	0.638	0.023	168.030
13	0.082	0.085	0.000	0.348	0.067	102.820
14	0.063	0.109	0.000	0.537	0.000	172.600
15	0.051	0.067	0.000	0.209	0.000	131.490
16	0.049	0.075	0.000	0.316	0.000	151.210
17	0.086	0.128	0.000	0.551	0.000	148.710
18	0.072	0.088	0.000	0.259	0.000	122.060

19	0.051	0.101	0.000	0.471	0.000	195.940
20	0.050	0.096	0.000	0.466	0.000	192.500
21	0.048	0.135	0.000	0.721	0.000	283.500
22	0.055	0.082	0.000	0.255	0.000	149.940
23	0.047	0.100	0.000	0.526	0.000	215.230
24	0.078	0.118	0.000	0.388	0.000	152.290
25	0.060	0.163	0.000	0.834	0.000	272.740
26	0.063	0.148	0.000	0.744	0.000	235.450
27	0.025	0.084	0.000	0.481	0.000	339.150
28	0.046	0.101	0.000	0.456	0.000	218.850
29	0.046	0.086	0.000	0.309	0.000	187.430
CHUK						
1	0.040	0.073	0.000	0.293	0.000	181.890
2	0.023	0.034	0.000	0.130	0.000	147.490
3	0.043	0.063	0.000	0.221	0.000	148.320
4	0.076	0.144	0.000	0.614	0.037	190.180
5	0.039	0.060	0.000	0.237	0.000	152.970
6	0.048	0.080	0.000	0.322	0.000	168.790
7	0.053	0.089	0.000	0.417	0.021	167.880
8	0.067	0.107	0.000	0.429	0.018	159.410
9	0.038	0.069	0.000	0.283	0.000	182.610
10	0.035	0.054	0.000	0.159	0.000	156.510
11	0.021	0.059	0.000	0.283	0.000	281.240
12	0.019	0.038	0.000	0.130	0.000	201.500
13	0.049	0.075	0.000	0.318	0.000	154.820
14	0.028	0.044	0.000	0.136	0.000	154.720
15	0.052	0.151	0.000	0.737	0.000	293.120
16	0.031	0.046	0.000	0.142	0.000	149.090
17	0.030	0.072	0.000	0.326	0.000	235.670
18	0.074	0.110	0.000	0.344	0.000	148.200
19	0.022	0.051	0.000	0.189	0.000	230.650
20	0.046	0.068	0.000	0.257	0.000	148.210
21	0.023	0.045	0.000	0.143	0.000	198.000
22	0.017	0.049	0.000	0.202	0.000	296.860
23	0.039	0.077	0.000	0.243	0.000	198.230
24	0.051	0.126	0.000	0.595	0.000	247.860
25	0.051	0.126	0.000	0.456	0.000	245.880
26	0.035	0.073	0.000	0.278	0.000	209.980
27	0.016	0.040	0.000	0.134	0.000	242.790
28	0.042	0.083	0.000	0.249	0.000	195.720
29	0.036	0.072	0.000	0.247	0.000	201.350

CHA: French charolais
CHCU: Charolais de Cuba
CHUK: British charolais
BTA: Chromosomes
F_{ROH_BTA}: Inbreeding per chromosomes and cattle populations calculated as the proportion of BTA in ROH over the length of the BTA covered by the involved SNPs.
±SD: Standard deviation.
Min: Minimum.
Max: Maximum.
CV(%):Coefficient of variation.

Table S2: Distribution of the number and size of runs of homozygosity (ROH) by po

BTA	CHA			CHCU		
	Number	Freq (%)	MN_{ROH_BTA} Mb	Number	Freq (%)	MN_{ROH_BTA} Mb
1	6	2.7	7.122	60	9.8	8.926
2	8	3.6	6.764	24	3.9	8.56
3	19	8.5	7.965	21	3.4	8.262
4	9	4	7.803	28	4.6	10.211
5	8	3.6	10.449	15	2.4	11.859
6	13	5.8	11.08	19	3.1	9.607
7	11	4.9	6.249	22	3.6	7.947
8	9	4	5.796	19	3.1	15.354
9	11	4.9	8.581	26	4.2	9.962
10	11	4.9	6.293	29	4.8	10.355
11	9	4	7.268	25	4.1	8.893
12	3	1.3	6.098	28	4.6	12.002
13	5	2.2	6.204	37	6.1	7.475
14	16	7.2	7.897	23	3.7	9.323
15	5	2.2	13.833	22	3.6	7.925
16	5	2.2	12.349	23	3.7	6.935
17	7	3.1	10.14	23	3.7	11.245
18	5	2.2	11.093	25	4.1	7.627
19	3	1.3	6.25	16	2.6	8.231
20	9	4	7.021	12	1.9	11.917
21	8	3.6	6.149	10	1.6	13.478
22	8	3.6	5.959	17	2.7	7.865
23	5	2.2	5.315	14	2.3	6.949
24	6	2.7	7.907	18	2.9	10.808
25	6	2.7	6.586	9	1.4	11.406
26	5	2.2	5.611	15	2.4	8.673
27	4	1.8	9.427	6	1	7.479
28	2	0.9	5.6	10	1.6	8.558
29	5	2.2	4.474	12	1.9	7.831

Number: ROH number found on each chromosome.

Freq: ROH percentage on each chromosome.

MN_{ROH_BTA}: average ROH size in Mb per chromosome.

Table S3: Value estimated of F_{ROH} in other cattle breeds.

Breed	n	F_{ROH}(±SD)	Country	Reference
**Brown Swiss**	304	0.074 ± 0.029[h]	Austria	[5]
**Fleckvieh**	502	0.019 ± 0.015[h]		
**Norwegian Red**	498	0.035 ± 0.021[h]		
**Tyrol Grey**	117	0.036 ± 0.031[h]		
**Holstein**	5.853	0.038 ± 0.021	UUEE	[40]
**Gyr**	167[a]<br>285[b]	0.232<br>0.035	Brazil	[44]
	2345[c]<br>563[d]	0.017 – 0.023[i]		[68]
**Sukuma**	40	0.005 ± 0.01	Tanzania	[41]
**Tarime**	40	0.009 ± 0.03		
**Maasai**	40	0.023 ± 0.05		
**Boran**	40	0.012 ± 0.01		
**Friesian**	40	0.018 ± 0.03		
**Blanc Bleu Belge**	634[e]	0.151 (0.098-0.237)[i]	Belgium	[42]
**Retinta**	32[f]	0.15 ± 0.091	Spain	[10]
	22[g]	0.04 ± 0.059		
**Spanish Lidia**	284	0.21-0.24[i]	Spain	[43]
**Brown**	749	0.097 (0.002-0.203) [i]	Italy	[31]
**Holstein**	2093	0.073 (0.006-0.233) [i]		
**Piedmontese**	364	0.046 (0.001-0.341) [i]		
**Marchigiana**	410	0.011 (0.000-0.124) [i]		
**Simmental**	479	0.028 (0.000-0.118) [i]		
**Cinisara**	71	0.052 ± 0.064	Italy	[27]
**Modicana**	72	0.055 ± 0.053		
**Italian Holstein**	96	0.042 ± 0.023		
**Reggiana**	168	0.035 ± 0.040		

[a] Beef population [b] Dairy population [c] females, [d] males, [e] sires, [f] Highly inbreed animals group (F_{PED}=0.164; 0.103-0.306), [g] Low inbreeding animals group (F_{PED}=0.08; 0- 0.025), [h] $F_{ROH>8}$ Mb; [i] range.
